# Evaluating spatial coverage of data on the aboveground biomass in undisturbed forests in the Brazilian Amazon

**DOI:** 10.1186/s13021-019-0126-8

**Published:** 2019-09-03

**Authors:** Graciela Tejada, Eric Bastos Görgens, Fernando Del Bon Espírito-Santo, Roberta Zecchini Cantinho, Jean Pierre Ometto

**Affiliations:** 1 Earth System Science Center (CCST), National Institute for Space Research (INPE), Av dos Astronautas 1758, São José dos Campos, SP 12227-010 Brazil; 2 Department of Forestry Engineering, Universidade Federal dos Vales do Jequitinhonha e Mucuri, Campus JK, Rod. MGT 367, km 583 5000, Alto do Jacuba, Diamantina, MG 39100-000 Brazil; 3 Centre for Landscape and Climate Research (CLCR) and Leicester Institute for Space and Earth Observation (LISEO), School of Geography, Geology and Environment, University of Leicester, University Road, Leicester, LE1 7RH UK; 4 United Nations Development Programme (UNDP), SEN 802, 17, Conj. C-St. Mans̃oes DB, Brasília, DF 70800-400 Brazil

**Keywords:** Amazon, Tropical rain forest, Remote sensing, Carbon, Aboveground biomass, REDD+

## Abstract

**Background:**

Brazilian Amazon forests contain a large stock of carbon that could be released into the atmosphere as a result of land use and cover change. To quantify the carbon stocks, Brazil has forest inventory plots from different sources, but they are unstandardized and not always available to the scientific community. Considering the Brazilian Amazon extension, the use of remote sensing, combined with forest inventory plots, is one of the best options to estimate forest aboveground biomass (AGB). Nevertheless, the combination of limited forest inventory data and different remote sensing products has resulted in significant differences in the spatial distribution of AGB estimates. This study evaluates the spatial coverage of AGB data (forest inventory plots, AGB maps and remote sensing products) in undisturbed forests in the Brazilian Amazon. Additionally, we analyze the interconnection between these data and AGB stakeholders producing the information. Specifically, we provide the first benchmark of the existing field plots in terms of their size, frequency, and spatial distribution.

**Results:**

We synthesized the coverage of forest inventory plots, AGB maps and airborne light detection and ranging (LiDAR) transects of the Brazilian Amazon. Although several extensive forest inventories have been implemented, these AGB data cover a small fraction of this region (e.g., central Amazon remains largely uncovered). Although the use of new technology such as airborne LiDAR cover a significant extension of AGB surveys, these data and forest plots represent only 1% of the entire forest area of the Brazilian Amazon.

**Conclusions:**

Considering that several institutions involved in forest inventories of the Brazilian Amazon have different goals, protocols, and time frames for forest surveys, forest inventory data of the Brazilian Amazon remain unstandardized. Research funding agencies have a very important role in establishing a clear sharing policy to make data free and open as well as in harmonizing the collection procedure. Nevertheless, the use of old and new forest inventory plots combined with airborne LiDAR data and satellite images will likely reduce the uncertainty of the AGB distribution of the Brazilian Amazon.

## Background

The Amazon forest is a region of great interest for biodiversity, conservation, and ecosystem services. The Amazon holds a large stock of carbon in undisturbed forest. However, land use and land cover change have greatly impacted these forests [1–3]. The carbon stock of undisturbed forests is the starting point for quantifying the carbon emissions from deforestation [4, 5].

To quantify the carbon stocks at the national scale, Amazon countries have been using forest inventory plots to measure aboveground biomass (AGB) [6, 7]. In the past few years, several studies have used high-resolution remote sensing data to estimate carbon stocks (e.g., Peru [8], Ecuador [9], Brazil [10–12]). AGB data estimates are also necessary for National Communications on greenhouse gases (GHG) and reduce emissions from deforestation and degradation (REDD+), both under the United Nations Framework Convention on Climate Change (UNFCCC) [13].

Brazil, which contains 60% of the Amazon region, has been using forest inventory plots to report its GHG inventories under the UNFCCC [6, 14, 15]. AGB quantification has many challenges, such as accessibility, long distances and high costs of field measurements in large areas, such as the Brazilian Amazon biome (~ 3,139,172 km^2^ of undisturbed forest [16]) [17]. There are many forest inventory plots with AGB field measurements [4]. However, the collected AGB data are unstandardized and not always available to the scientific community to quantify forest carbon stocks.

Given the great extent and variability of forest structures in the tropics, remote sensing is one of the best tools for estimating the AGB [18, 19] of tropical forests. With the new remote sensing sensors and statistical methods, such as light detection and ranging (LiDAR) and random forest interpolation modeling, there has been a great advance in the AGB estimates [20, 21] in the Brazilian Amazon. However, these efforts are still limited by the availability of data derived from field forest inventories [17, 22]. The combination of field AGB data and different remote sensing products has resulted in significant differences in the spatial distribution of AGB estimates in produced AGB maps of the Brazilian Amazon [22, 23]. As a result, in estimating carbon emissions from deforestation, forest AGB remain the largest source of uncertainty in the tropics [5, 23].

This study evaluates the spatial coverage of AGB data in undisturbed forests in the Brazilian Amazon. We present the location and characteristics of forest inventory plots, AGB maps and remote sensing products. In addition, we analyzed the interconnection between these data and stakeholders generating the data (national forest inventories, ecological networks, projects and institutions). We identified the fraction of the undisturbed forest covered by forest inventories and evaluated the distribution of forest inventory plots across environmental factor maps (soil, topography, vegetation and climate).

## Methods

**Fig. 1:**
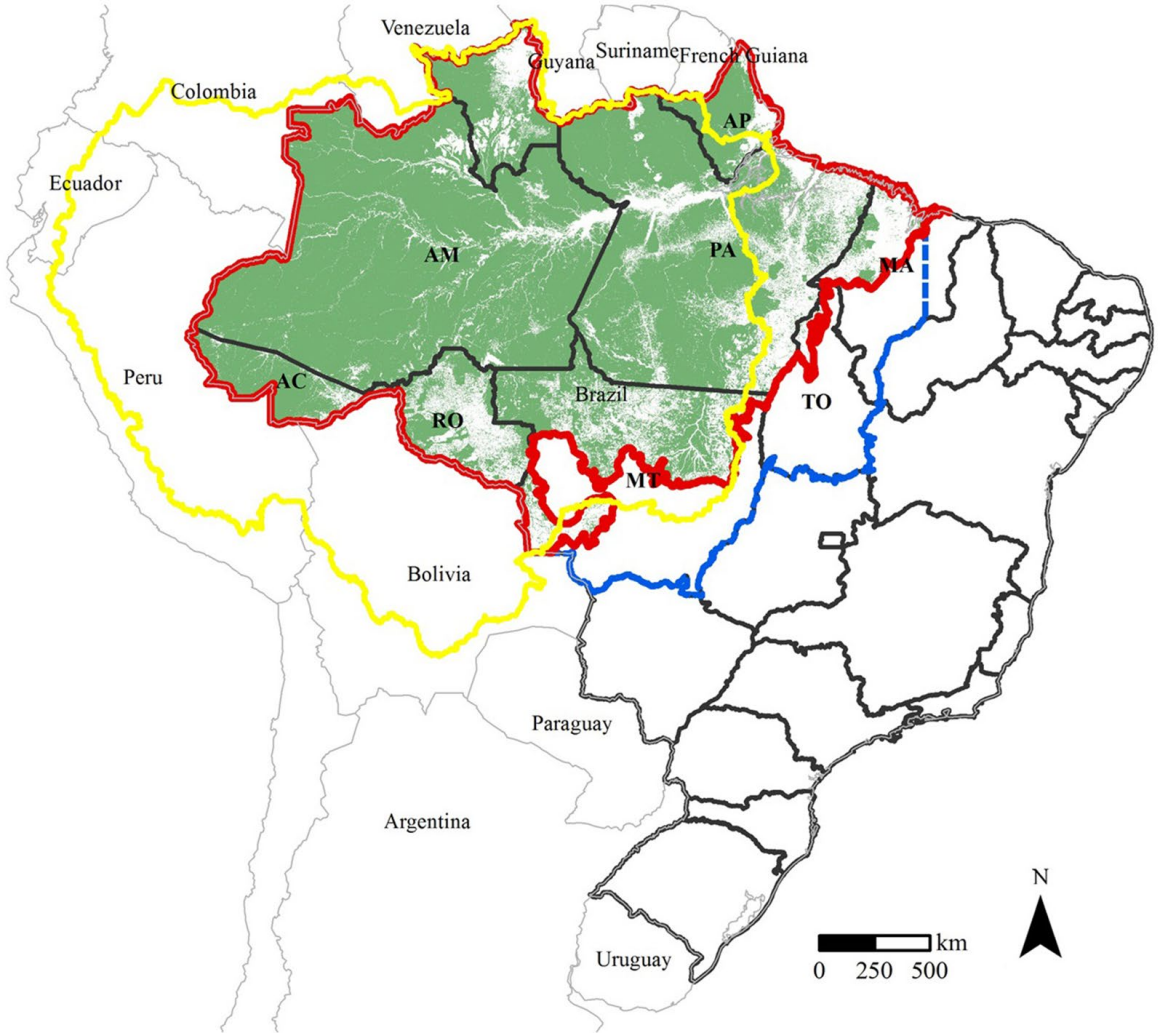
Spatial distribution of forests in the Brazilian Amazon biome, our study area. Brazilian Amazon biome forests, our study area (red line). The boundaries of the Brazilian Legal Amazon (blue line) and Amazon Basin (yellow line) are also shown. The 2014 forest mask data are from PRODES [16] (green) and the Brazilian biomes data are from IBGE [24]. The Brazilian states of Acre, Amazonas, Amapá, Mato Grosso, Maranhão, Pará, Rondônia, Roraima, and Tocantins are represented by AC, AM, AP, MT, MA, PA, RO, and TO, respectively

This study focused on undisturbed forests of the Brazilian Amazon biome, an area of approximately 3,139,172 km^2^, considering the 2014th deforestation mask provided by the Deforestation Monitoring Program (PRODES) data [16, 24] (Fig. 1).

The results derived from this study were cohesive derived from the following: [1] review and organization of the existing AGB data, i.e., forest inventory plot locations, airborne LiDAR transects and AGB maps across the Brazilian Amazon biome; [2] social network analysis (SNA) of the stakeholders working with AGB data; [3] coverage of forest inventory plots; and [4] quantification of the forest inventory plots across environmental factor maps (soil, topography, vegetation and climate).

We reviewed and organized the available AGB datasets of the Brazilian Amazon, e.g., past and ongoing forest inventory data, published AGB maps (and the field data used to produce them), airborne LiDAR transects and environmental factor maps. We used the following criteria to consider a forest inventory dataset: (i) the data are from undisturbed forests in the Amazon biome; (ii) the data must originate on more than one site in the Brazilian Amazon biome; and (iii) the forest inventory stakeholders have many interinstitutional collaborations. Several field datasets were available from personal contacts [25–30]. All datasets from available AGB maps [6, 15, 18, 22, 31–35] and field plot locations were organized in a georeferenced dataset, and their institutional relations were placed in a Table as input to make an SNA (Additional file 1: Table S1).

We used an SNA to identify the relation between the stakeholders of field plots and AGB maps. The SNA consists of a set of actors (called nodes), a set of connections (called edges or links) between the actors, and an attribute that describes the type of each actor [36]. In our analyses, the actors were the stakeholders working with AGB data in the Brazilian Amazon biome. The attributes were the type of stakeholders, i.e., national or international universities, projects, main sites, main networks and institutions. The connections were the collaborations and links between the stakeholders (e.g., sharing field or remote sensing data) and were counted in pairs of actors, where one actor could have one or many connections (a detailed Table of the SNA is provided in Additional file 1: Table S1). The output is usually a figure that represents the connection strength between stakeholders; each stakeholder is a box, and the larger the boxes are (more connections), the stronger the connections. The connections are represented by lines, and the attributes of the stakeholders are denoted by the color of the box.

To quantify the coverage of the AGB field plot data, we calculated the distance from the forest inventory plots in the Brazilian Amazon forest. To estimate the sampled area of the AGB plots, we considered the reported area of each forest inventory dataset. The location and area of LiDAR surveys were from two leading projects: the improving biomass estimation methods for the Amazon (EBA) [37] and sustainable landscapes (SL) [27].

We evaluated the representativeness of the forest inventory datasets by calculating the number of plots in each environmental factor map: soil with 42 classes [38], topography with 31 classes [39], vegetation with 28 classes [15] and climate expressed as dry months with 5 classes [40].

## Results

### AGB datasets

#### Forest inventories

**Fig. 2:**
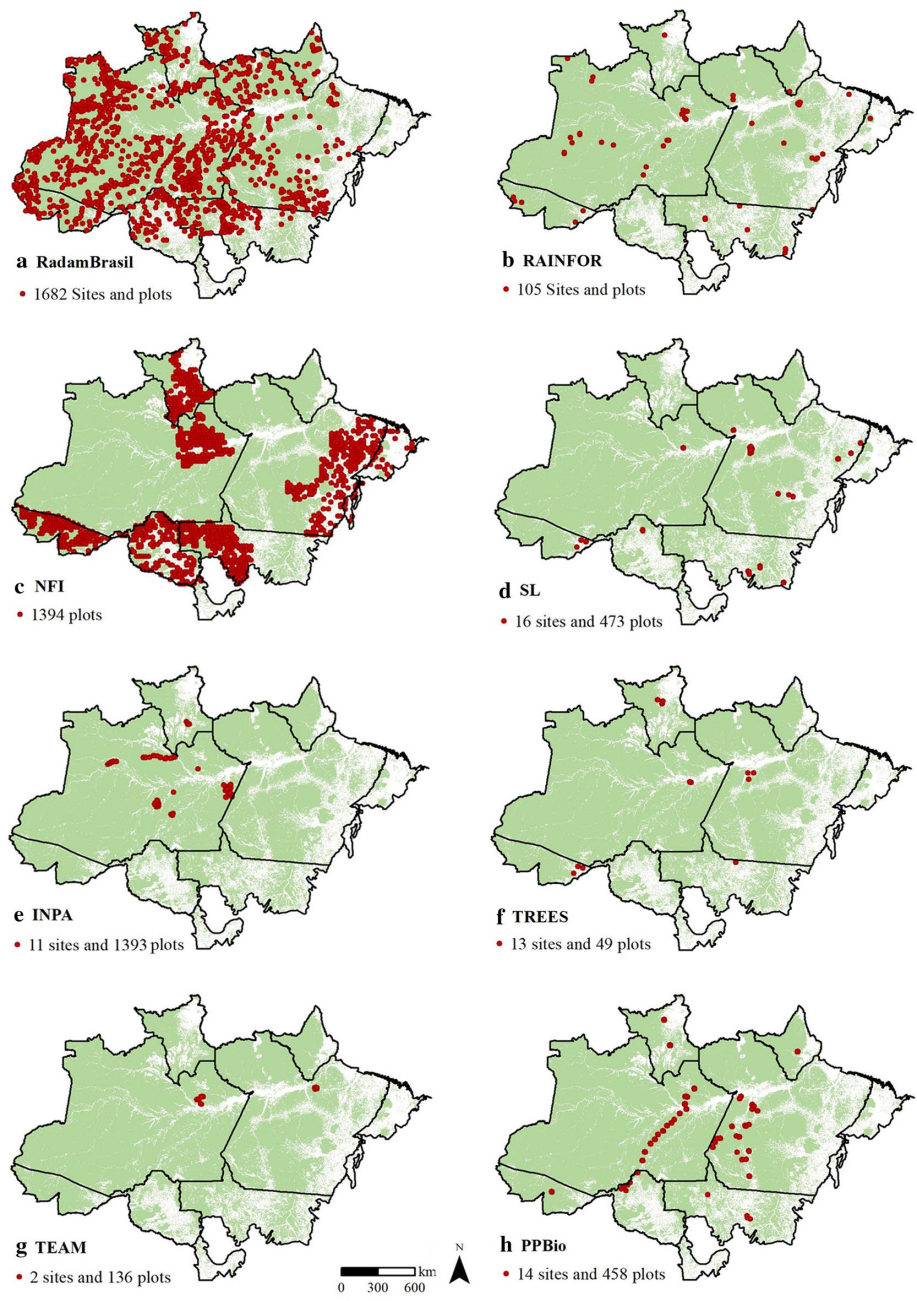
Distribution of forest inventory plots in the Brazilian Amazon. **a** RadamBrasil [41]; **b** Amazon Forest Inventory Network (RAINFOR) [75]; **c** National Forest Inventory [26]; **d** sustainable landscapes project [27]; **e** National Institute of Amazon Research (INPA) (personal communication); **f** Tropical Ecosystems and Environmental Sciences Laboratory (TREES) [30]; **g** Tropical Ecology, Assessment and Monitoring Network (TEAM) [42]; and **h** Research Program for Biodiversity (PPBio) [45]

We found at least ten stakeholders working on forest inventory plots of the Brazilian Amazon (Table 1). Each stakeholder sampled the forests using different protocols (i.e., objectives, plot sizes, area, spatial coverage, and sites). The largest forest inventory is from RadamBrasil (n = 1682 plots of 1 ha), which was sampled between 1973 and 1980. Six of the current AGB stakeholders of the Brazilian Amazon [the Amazon Forest Inventory Network (RAINFOR); Tropical Ecology, Assessment and Monitoring (TEAM); the Research Program for Biodiversity (PPBIO); SL; the Brazilian Forest Service; and the Tropical Ecosystems and Environmental Sciences Laboratory (TREES)] are still collecting forest inventory data in permanent plots (Fig. 2).

**Table 1:** Description of the forest inventory plots of the Brazilian Amazon (institutions, networks and projects)

Stakeholders	Scale	Objective of AGB collection	Initial measurements/remeasurements	Total plots/Brazilian plots	Plots in the study area/sampled area (ha)	Carbon pools measured	Availability	Web page
Amazon forest inventory network (RAINFOR)	Amazon Basin	Monitor large-scale patterns of forest structure and dynamics across Amazonia	~ 1960/yes	413/141	105/405	AGB	Yes, online	http://www.forestplots.net/
RadamBrasil	Brazilian Amazon	Large-scale forest inventories aiming at commercial trees	1973–1983/no	2702/2702	1682/1682	AGB	Yes, online	http://sirene.mcti.gov.br
Tropical ecology assessment and monitoring (TEAM) Network	Pantropical	Monitor long-term trends in biodiversity, land cover change, climate and ecosystem services in tropical forests	2002/yes	1021/136	136/136	AGB	Yes, online	http://www.teamnetwork.org/
Research program for biodiversity (PPBio)	Brazil	Intensify biodiversity studies in Brazil, decentralizing the scientific production to disseminate the results	2004/yes	> 1000/ND	458/458	AGB	Yes, online	http://ppbio.inpa.gov.br/repositorio/dados
Sustainable landscapes	Brazilian Amazon/local (São Paulo, Santa Catarina)	Focus on airborne LiDAR and degraded forests, using field plots to calibrate the empirical relations between ALS and AGB	2012/yes	> 500	473/115	AGB	Yes, online	https://www.paisagenslidar.cnptia.embrapa.br/webgis//
INPA-Amazonas state forest inventory	Regional (Amazonas state), local (Acre, Pará, Roraima)	Establish a continuous forestry inventory system of Amazonas state	1980/yes	ND/2503	1362 plots/1362	AGB, few trees of BGB	No^b^	https://www.inpa.gov.br
Brazilian forest service								
National Forest Inventory	Brazil	Generate information on forest resources (natural and plantations) every 5 years	2013–2017/yes	10,091 (of 17,580 planned)/10,091	1202 (of 5828 planned)/240	AGB, litter, soil, dead wood	Not yet for the Amazon biome, yes for the rest	http://ifn.florestal.gov.br/
Permanent plots in forest concessions	Local (Rondônia and Pará)	Monitor forest concessions	2010	192	192/^a^38.4	AGB	ND	http://www.florestal.gov.br/monitoramento
Redeflor	Brazil	Monitor forest dynamics through permanent plots	ND	800	ND/ND	ND	No	http://redeflor.net/
Tropical ecosystems and environmental Sciences Laboratory (TREES)	Local (Acre, Rondônia, Alta floresta, Pará, Manaus)	Assess the impacts of environmental changes on tropical ecosystems using remote sensing and field surveys, with focus on fire	2012/yes	60	49/17	AGB	Yes, through RAINFOR site	http://trees-research.weebly.com/

*ND* no data, *AGB* aboveground biomass, *BGB* belowground biomass, *ALS* airborne laser scanning, *LiDAR* light detection and ranging

^a^In the case of the sampled area of the forest concessions, we assumed that the area was the same as the of the National Forest Inventory (0.2 ha)

^b^The biomass and carbon data of the plots of the National Forest Inventory for the states of the Amazon biome are not yet available online, although the data are available for other states that have already ceased collecting measurements

The RadamBrasil project (1973–1983) recorded 2702 plots considering only commercial trees [41]. This dataset remains widely used due to its extensive coverage despite the date of measurement (almost 30 years ago) and absence of remeasurements. RadamBrasil plots were the field data input for the biomass maps of the second and third National Communications of Brazil to the UNFCCC [26, 27] and Nogueira et al. [30, 34] (Fig. 2a, c).

INPA’s Forest Management Laboratory maintains an extensive plot network that includes the Continuous Forest Inventory (CFI) of the Amazonas state and contains more than 2500 AGB plots. Some of the plots are included in the RAINFOR, PPBio and TEAM [42].

The RAINFOR network monitors 413 AGB plots in the Amazon Basin, of which 105 are located in the Brazilian Amazon biome (Fig. 2b) [14, 15]. The TEAM network has two sites in the Brazilian Amazon, one in Manaus and the other in Caxiuanã, including a total of 136 AGB plots (Fig. 2g).

The TREES of the National Institute for Space Research (INPE) has 49 plots, of which 17 are used to monitor AGB (Fig. 2f) (the other plots are used to monitor fire impacts). The AGB plots are available through the RAINFOR website.

The SL project has airborne laser scanning (ALS) data and to calibrate remote sensing-based models, they monitor 473 AGB plots [32, 43]. Some of the AGB plots being monitored are part of other stakeholders (e.g., Embrapa Acre). All the recorded plots and the ALS dataset are recent, and the data are completely available on the Internet.

Another network is Redeflor, with 794 permanent plots around the Amazon [44]. The spatial locations of the plots are not available. The Brazilian Agricultural Research Corporation (Embrapa), universities, and some forest companies are part of the Redeflor forest inventory. Many of the Redeflor plots are included in the SL forest inventory.

INPA holds the international PPBio program. This program gathers many universities and institutes with the objective of decentralizing biodiversity studies and disseminating the results of biodiversity data. PPBio has approximately 460 1-ha plots in the Brazilian Amazon biome [45, 46].

The Brazilian Forest Service is in charge of the National Forest Inventory (NFI), for which extensive and systematic sampling is performed over a 5 × 5 km grid. As of February 2019, 2280 (1202 in intact forest areas) out of 5828 planned sample plots had already been recorded in the Brazilian Amazon biome. Each plot is 0.2 ha. However, it is not clear how the NFI data will be published and distributed for the Amazon biome, although for the rest of the Brazilian biomes, AGB data are available online (Fig. 2c) [47]. The small size of the NFI plots brings abundant controversy regarding the best plot size for carbon assessments [48–50]. In addition to the NFI, the Brazilian Forest Service also has 192 permanent plots in forest areas under concession (Fig. 2c) [26].

#### Remote sensing data

The main remote sensing products of the vegetation index at the global level are Vegetation Tree Cover [11], GlobCover 2009 [51] and GLC 2000 [52]. These products are mainly based on optical datasets, such as those for Landsat and MODIS. The combination of Landsat and MODIS, active sensors from satellite platforms, such as Geoscience Laser Altimeter System (GLAS)-LiDAR, and forest inventory plots are used to generate AGB maps at a pantropical scale [18, 33], as shown in Table 2. Remote sensing technologies allow the estimation of forest biomass even over extensive and inaccessible areas. Airborne LiDAR and radar allow forest structure estimates in 3 dimensions [20], which is highly recommended for AGB inventories [19, 53].

**Table 2:** Main characteristics of the Amazon forest AGB density maps

Map	Scale	Spatial resolution	Temporal scale (years)	Field forest plots/source	Study area plots/sampled area (ha)	Remote sensing products/other inputs	Model
Saatchi et al. [31]	Amazon Basin	1 km	2000–2004	544/many sources	~ 361/~ 1633^d^	MODIS (NDVI, LAI, % tree cover), JERS-1 radar, SRTM/vegetation map, climate data (WorldClim)	Biomass classification approach
Nogueira et al. [32]	Brazilian Amazon	1 km (landscape level)	Only 1976	2879/RadamBrasil and literature	2879/2879	No/vegetation map [40]	None
MCT [15]	Brazilian Amazon	1 km (landscape level)	1973–1983^a^	1710^c^/RadamBrasil and literature	1682/1682	No/vegetation [19], soils [41]	None
Saatchi et al. [18]	Pantropical	1 km	2000	4079^b^ (493 for calibration)/many sources	~ 707/~ 1770^d^	MODIS (NDVI, LAI, % tree cover), LiDAR from GLAS/forest height map	MaxEnt
Baccini et al. [33]	Pantropical	500 m	2007–2008	283^b^/measured	No data	MODIS, LiDAR from GLAS, SRTM	RandomForest
Mitchard et al. [22]	Amazon Basin	500 m	1960–2013^a^	413/RAINFOR and TEAM	105/405	No/regional map based on geography and substrate origin	Kriging, inverse distance kernel
Nogueira et al. [34]	Brazilian Amazon	1 km (landscape level)	1970^a^	2317^c^/RadamBrasil and literature	2373/2317	No/vegetation map [40]	None
Avitabile et al. [35]	Pantropical	1 km	2000–2013^a^	648/RAINFOR, TEAM and sustainable landscapes	~500/No data	No/high-resolution AGB maps	Fusion model
MCT [6]	Brazilian Amazon	1 km (landscape level)	1973–1983^a^	1682 plots/RadamBrasil	1682/1682	No/vegetation [19], soils [41]	Inverse distance weighting

*RAINFOR* Amazon forest inventory network, *TEAM* tropical ecology, assessment and monitoring, *MODIS* moderate resolution imaging spectroradiometer, *NDVI* normalized difference vegetation index, *LAI* leaf area index, *GLAS* geoscience laser altimeter system, *LiDAR* light detection and ranging, *SRTM* shuttle radar topography mission, *JERS-1* Japanese earth resources satellite 1

^a^AGB field measurements

^b^We did not have access to the locations of the plots

^c^In the case of the RadamBrasil plots, we had the locations of only 1682 plots

^d^The total area of the plots was estimated because the plot had different sizes

**Fig. 3:**
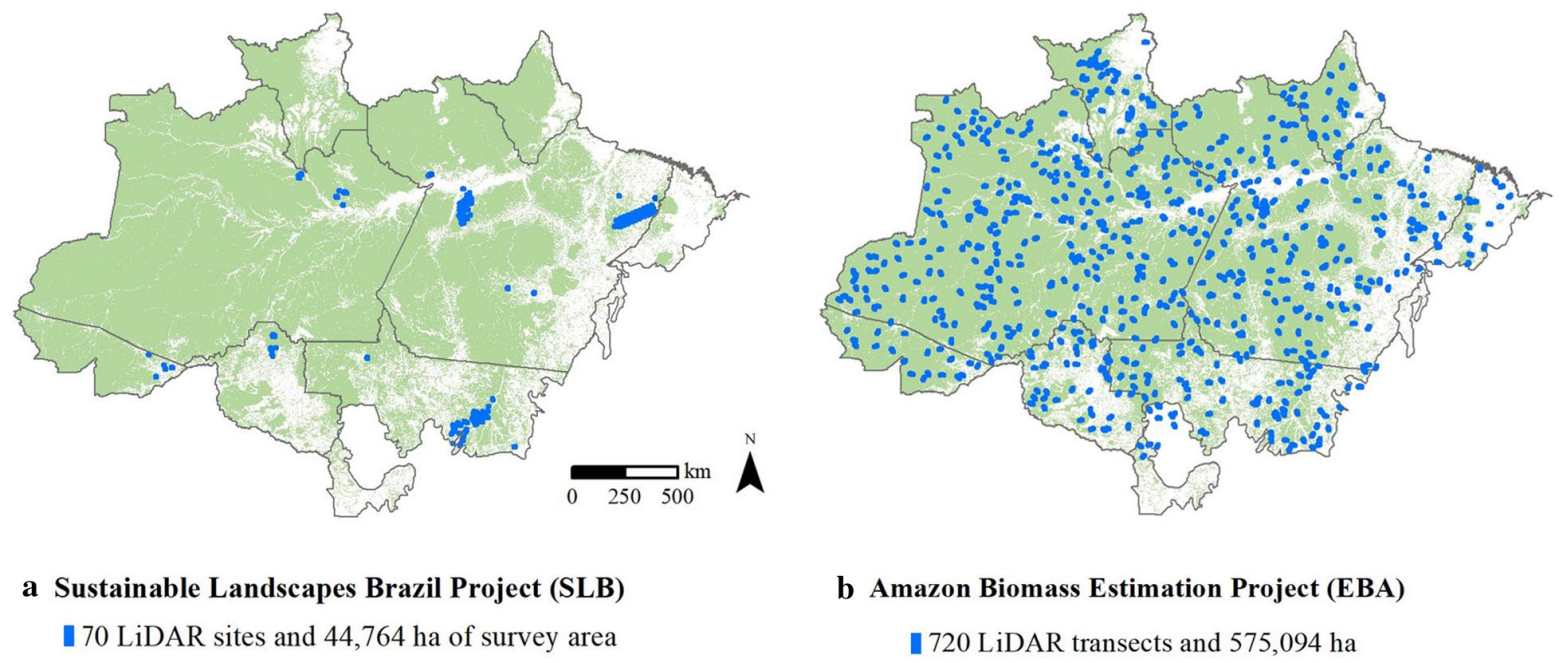
Distribution of the airborne LiDAR data in the Brazilian Amazon. **a** Sustainable landscapes [54]; **b** Amazon Biomass Estimation subproject 7 [37]

Two projects are currently working with airborne LiDAR. The SL project has been running ALS surveys in different biomes (available at: https://www.paisagenslidar.cnptia.embrapa.br/webgis//) [27, 54]. The total area surveyed over the Amazon biome reached 44,764 ha in 2017 and is still increasing (Fig. 3a). The EBA (Improving Biomass Estimation Methods for the Amazon) project has 720 transects (and 130 transects overflown) with a total of 575,094 ha surveyed. Some of the transects have airborne hyperspectral data. EBA does not have AGB plots and is going to use plots from partners for calibration and validation [37]. SL and EBA are now focused on assessing the AGB of forest areas under different conditions (degraded, secondary, primary, etc.).

#### Forest AGB maps

**Fig. 4:**
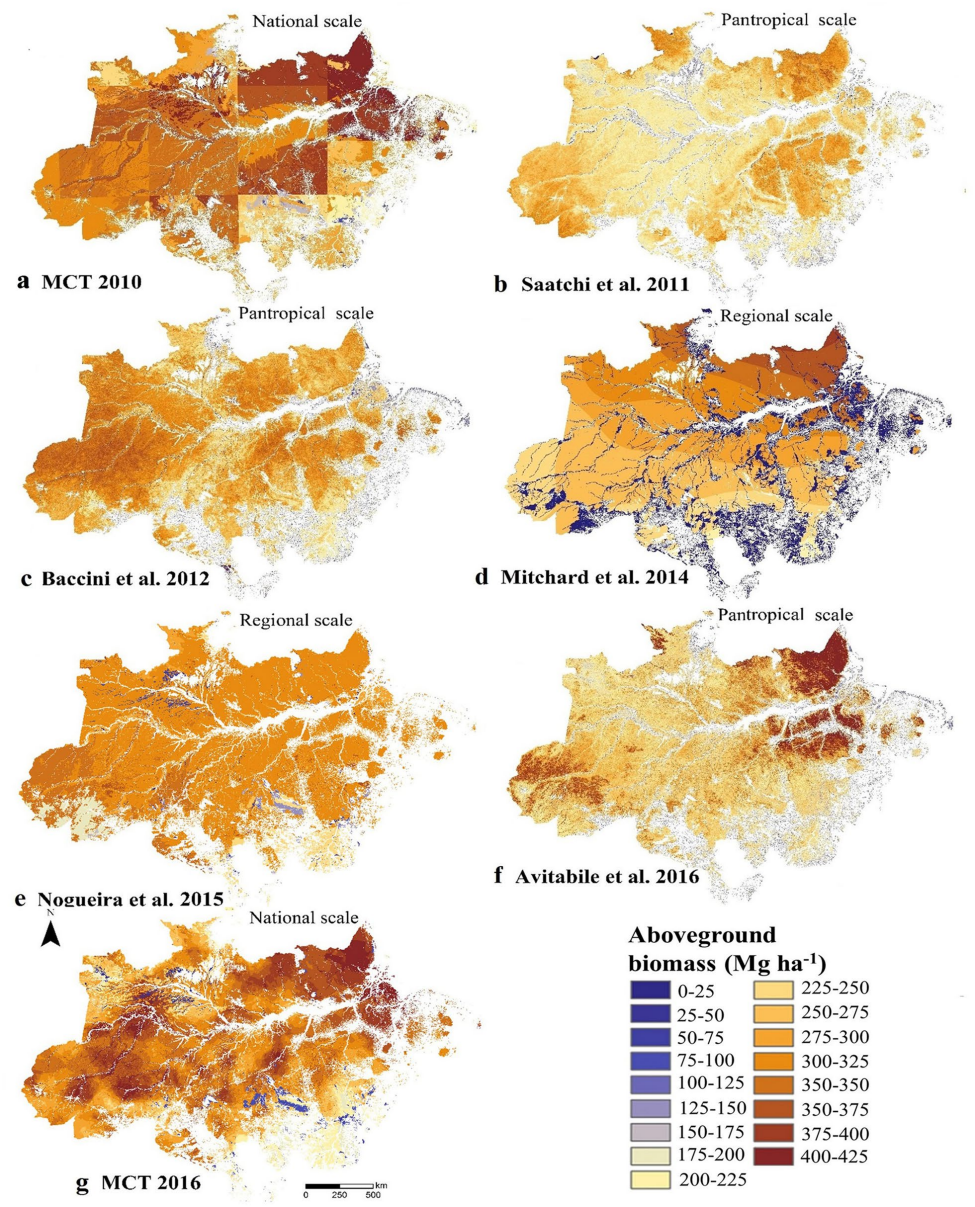
Spatial distribution of AGB maps in the Brazilian Amazon. The distributions of AGB were normalized for the same biomass ranges. All units are in megagrams per hectare

The AGB maps for the Brazilian Amazon show differences in both AGB quantity and distribution (Table 2). For example, the National Communications AGB maps differ among themselves (Fig. 4a, g). Part of the difference is due to the spatialization technique. The Second Brazilian National Communication map presented the AGB estimates as a result of the aggregation of the AGB values per vegetation class and extrapolated considering RadamBrasil volume sheets. This approach leads to a gross quadrant-like AGB distribution [23, 55]. For the third National Communication map, a combination of extrapolation methods, equations and expansion factors were used, returning completely different AGB estimates [6]. Nogueira et al. [32, 34] produced an AGB map employing RadamBrasil field data and a stratification approach aggregating AGB by vegetation map classes (Fig. 4c, Table 2).

At the pantropical scale, the map of Saatchi et al. [18] used a combination of global forest height, remote sensing, and field data (Fig. 4b, Table 2). It was employed as the basis for determining carbon emissions from the deforestation map of Harris et al. [56]. Another map constructed at the pantropical scale is the carbon density map of Baccini et al. [33] (Table 2 and Fig. 4c), which was based on multispectral surface reflectance data and established field plots colocated with satellite LiDAR footprints. Mitchard et al. [22] (Fig. 4d) produced an AGB map from a kriging extrapolation of RAINFOR forest inventory plots. Avitabile et al. [35] (Fig. 4f) combined 2 maps [18, 33] using a data fusion approach that included field data from RAINFOR and the SL project to produce a new AGB map (see Table 1).

#### Environmental factors

Environmental factors, such as climate, soil and topography, have been used for a wide range of AGB estimates in the Brazilian Amazon [31, 57, 58]. Our compilation of the environmental factors showed 13 layers available at the Amazon scale (Table 3).

**Table 3:** Environmental factor maps in the Brazilian Amazon

Environmental factor	Maps	Description	Coverage	Spatial resolution scale	Download site
Vegetation	Vegetation map [62]	Based on the RadamBrasil map, with the land-use classes updated by the SIVAM project	National	1: 250,000	http://mapas.mma.gov.br/i3geo/datadownload.htm
	IBGE vegetation map [63]	Part of the wall maps of IBGE, based on RadamBrasil map with the land-use classes updated by the SIVAM project	National	1: 5,000,000	ftp://geoftp.ibge.gov.br/informacoes_ambientais/
	Vegetation physiognomies of Brazil [15]	Map used in the National Communications grouping of the transition classes of the IBGE vegetation map [63]	Regional	1: 250,000	http://sirene.mcti.gov.br
Soils	Soil map of Brazil [64]	The soil map used in the new Brazilian system of soil classification of Embrapa and published by IBGE	National	1: 5,000,000	http://mapas.mma.gov.br/i3geo/datadownload.htm
	Soils of legal Amazon [65]	This is an adaptation of the Embrapa/IBGE 2001 soil map [64]	National	1: 250,000	http://mapas.mma.gov.br/i3geo/datadownload.htm
	Soils [38]	Soil carbon stocks	National	–	–
	Soil map [66]	Soil maps with particular reference to RAINFOR sites. Basin wide distributions of soils under forest vegetation	Regional	1: 5,000,000	–
Climate	WorldClim global climate data	WorldClim, uses meteorological field station observations from 1950 to 2000	Global	–	www.worldclim.org
	Climate map of Brazil [40]	Thematic map of Brazil, data from 1978 with adaptations in 2002	National	1: 5,000,000	http://www.ibge.gov.br/english/geociencias/default_prod.shtm
Elevation	SRTM 90 m (NASA, 2000)	SRTM of 90 m resolution	Global	90 m	http://www2.jpl.nasa.gov/srtm/cbanddataproducts.html
	SRTM 30 m (TOPODATA)	SRTM of 30 m resolution	Global	30 m	http://www2.jpl.nasa.gov/srtm/cbanddataproducts.html
Topography	Relief map 2002 [39]	Relief map 2002 (Compartimentos do relevo do Brasil—2002)	National	1: 250,000	http://mapas.mma.gov.br/i3geo/datadownload.htm
	Relief units map of Brazil [67]	Thematic map, based on the RadamBrasil Project and improved by the SIVAM project	National	1: 5,000,000	ftp://geoftp.ibge.gov.br/informacoes_ambientais/geomorfologia/vetores/brasil/

The representation of AGB is strongly associated with precipitation (both its amount and seasonality), which ranges from 80 to 300 mm/month. Additionally, the gradient in nutrient availability (mainly phosphorus) throughout the Amazon is also associated with AGB [59]. Vegetation physiognomy maps [60] have been used as inputs for many biomass maps [6, 14, 15, 34, 61].

### AGB stakeholders

**Fig. 5:**
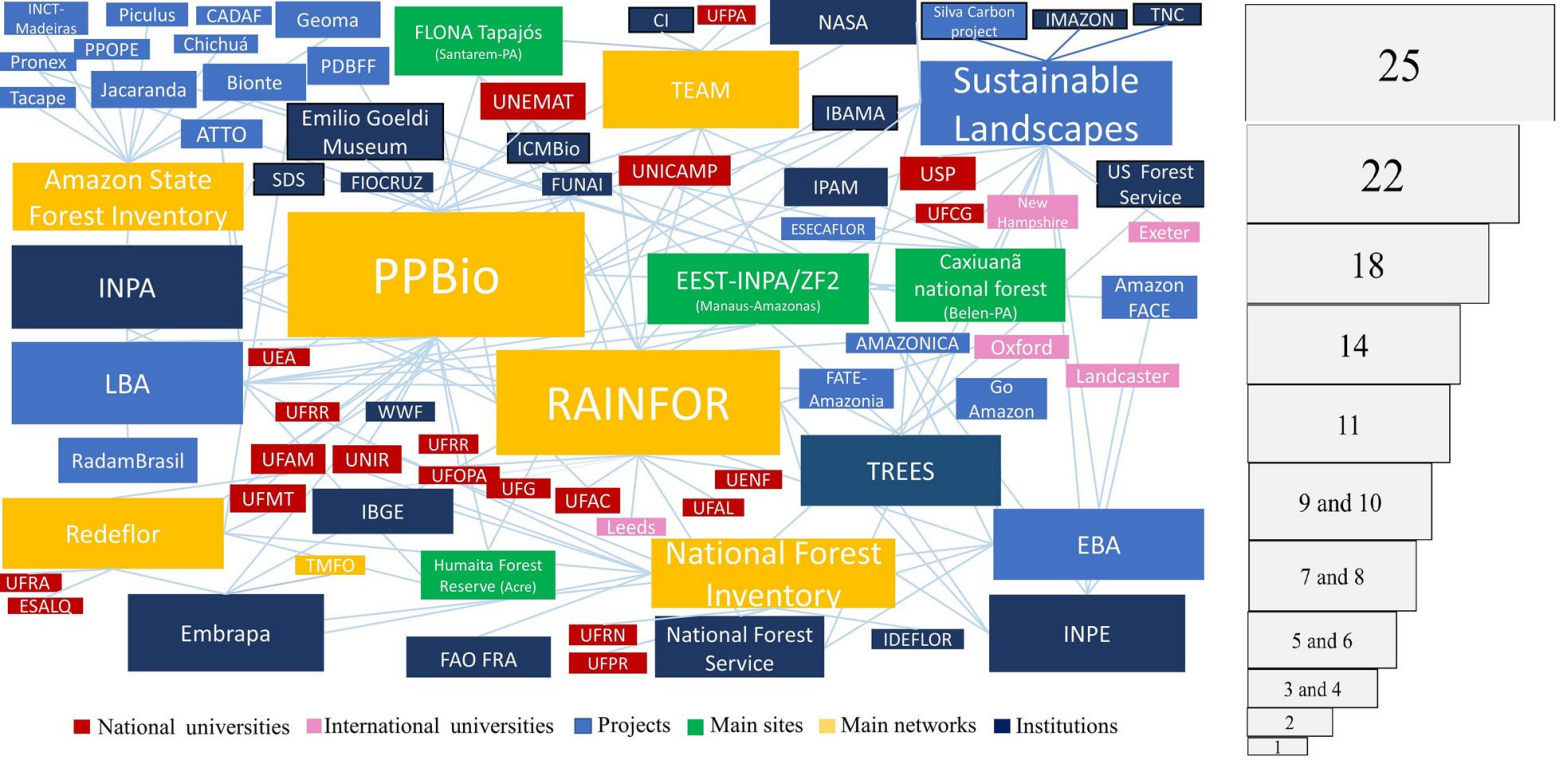
Connections between stakeholders of forest inventory plots of the Brazilian Amazon. Stakeholders include networks, projects, institutions, universities and sites. The size of each box represents the number of connections between the stakeholders. A Table of the SNA is provided in Additional file 1: Table S1 and contains detailed information regarding the connections and acronyms

The SNA results reveal the interrelationship between the different stakeholders working with AGB data. Stronger relationships are represented by the size of the box, and the more connections between the stakeholders, the larger the box size (Fig. 5); a detailed number of connections is provided in Additional file 1: Table S1.

The most connected stakeholders are the networks. PPBio has 9% of the total connections, followed by RAINFOR with 8%, both of which gather several institutions, universities, sites, projects and other networks. The SL project follows with 7% gathering national and international institutions, universities and networks (Fig. 5). The National and the Amazon state forest inventories also have many connections, 5% and 4%, respectively, although they are not connected to each other.

The large-scale biosphere–atmosphere experiment in Amazonia (LBA), known for its flux towers and AGB plots, is a project with many connections (4%) and has been collecting data since 1999. Institutions such as the INPA, INPE (holding the TREES laboratory and EBA project) and Embrapa are also visible stakeholders in Fig. 5, with more than 3% of the connections. Well-known sites with AGB plots are the ZF2 in Manaus (3%), Tapajós in Santarem (2%), and Caxiuanã in Belem (2%), which are shared by many networks, institutions and projects.

### Coverage of the forest inventory data

Taking into account the plots gathered by the forest inventory stakeholders, we found at least 5351 plots spread out over the Brazilian Amazon forest (Table 4). Among the plots, 26% are measured and maintained by INPA, with 26% of the current plots being attributed to the NFI and 25% to RadamBrasil, followed by SL (9%) and PPBio (9%). Other initiatives are responsible for less than 5%. We observed that the forest inventory plots area cover only 0.0013% of the total forest area of the Brazilian Amazon.

**Table 4:** Sampled area of forest inventory plots and LiDAR transects in the Brazilian Amazon forest biome

	Field plots									LiDAR transects		
	RadamBrasil	RAINFOR	SL	INPA	TREES	PPBio	NFI^a^	TEAM	Total	SL	EBA	Total
Plots per network	1362	105	473	1374	49	458	1394	136	5351	–	–	
LiDAR transects	–	–	–	–	–	–	–	–		70 sites	720	
% of plots from the total number of plots	25	2	9	26	1	9	26	3	100	–	–	
Area (ha)	1362	405	115	1374	17	458	279	136	4145	44,764	575,094	619,858
Total forest area (ha)											313,917,200	
% of area from the total forest area	0.00043	0.00013	0.00004	0.00044	0.00001	0.00015	0.00009	0.00004	0.00132	0.014	0.183	0.197
% of total area (plots and LiDAR)	0.20											

The number of plots for INPA, PPBio and RadamBrasil refers to those with location information. In the case of the NFI, are those measured or in the process of measurement and 192 plots of forest concessions

*RAINFOR* Amazon forest inventory network, *SL* sustainable landscapes, *TEAM* tropical ecology, assessment and monitoring, *INPA* National Institute of Amazon Research, *PPBio* research program for biodiversity, *TREES* Tropical Ecosystems and Environmental Sciences Laboratory, *NFI* National Forest Inventory, *EBA* improving biomass estimation methods for the Amazon

^a^We assume the plot sizes of the NFI (0.2 ha) for the plots of the forest concessions

**Fig. 6:**
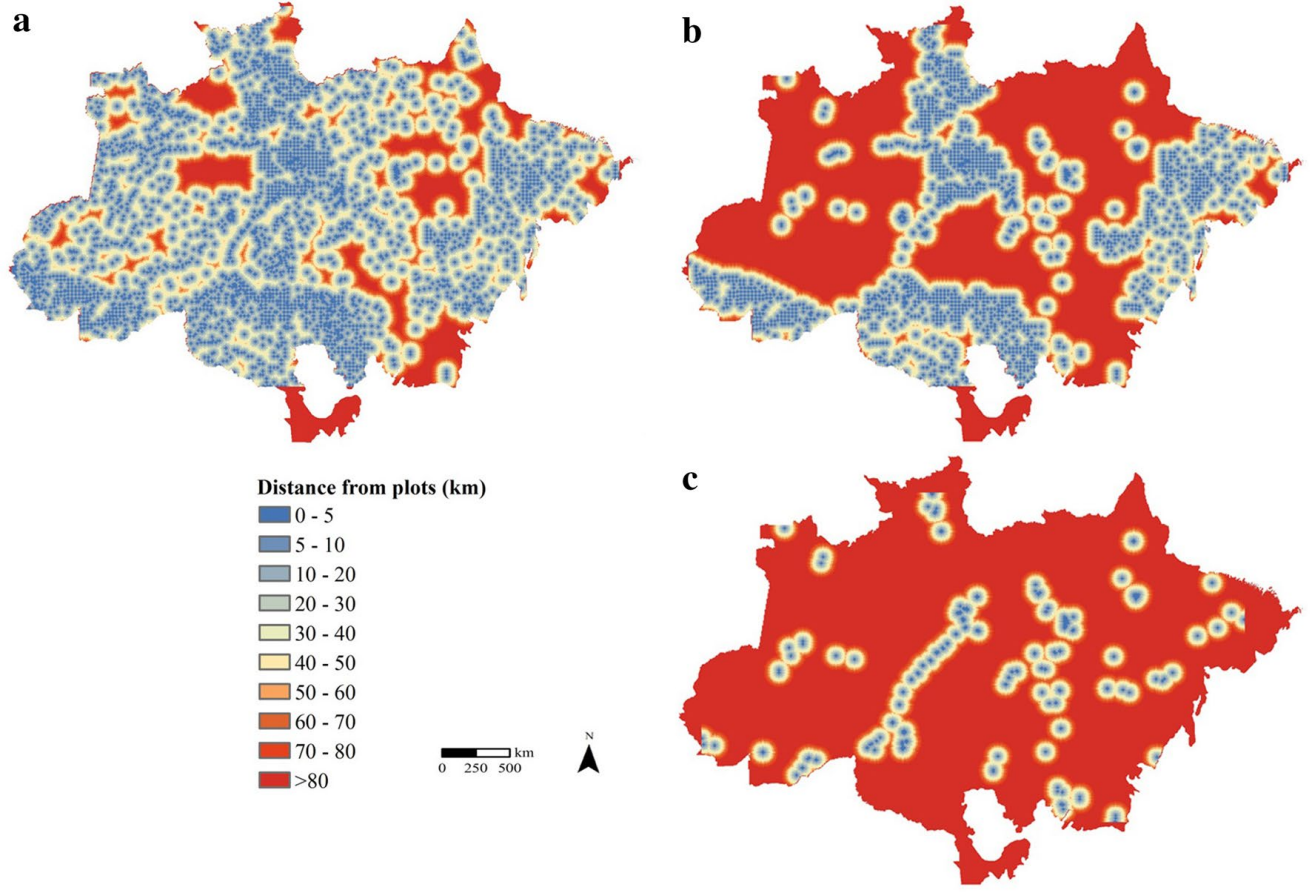
Distances from forest inventory plots in the Brazilian Amazon. **a** Considering all forest inventory plots; **b** excluding plots from RadamBrasil; and **c** excluding the data from RadamBrasil, INPA and the National Forest Inventory plots

The distance from the current plots (considering all forest inventory plots) is shown in Fig. 6a. The area of the Brazilian Amazon biome with more than 50 km from the nearest plot is 708,600 km^2^, representing 17% of the total area. In Fig. 6b, we show the distribution of the AGB dataset without RadamBrasil (for being old measurements), indicating a large increase (2,246,500 km^2^) in the places with no plot data, which represent 42% of the total area. Figure 6c presents the situation without the RadamBrasil, NFI (not yet available) and INPA (not available) plots, showing that more than 80% (3,409,750 km^2^) of the Brazilian Amazon biome has no plot representativeness.

### AGB data and environmental factors

**Fig. 7:**
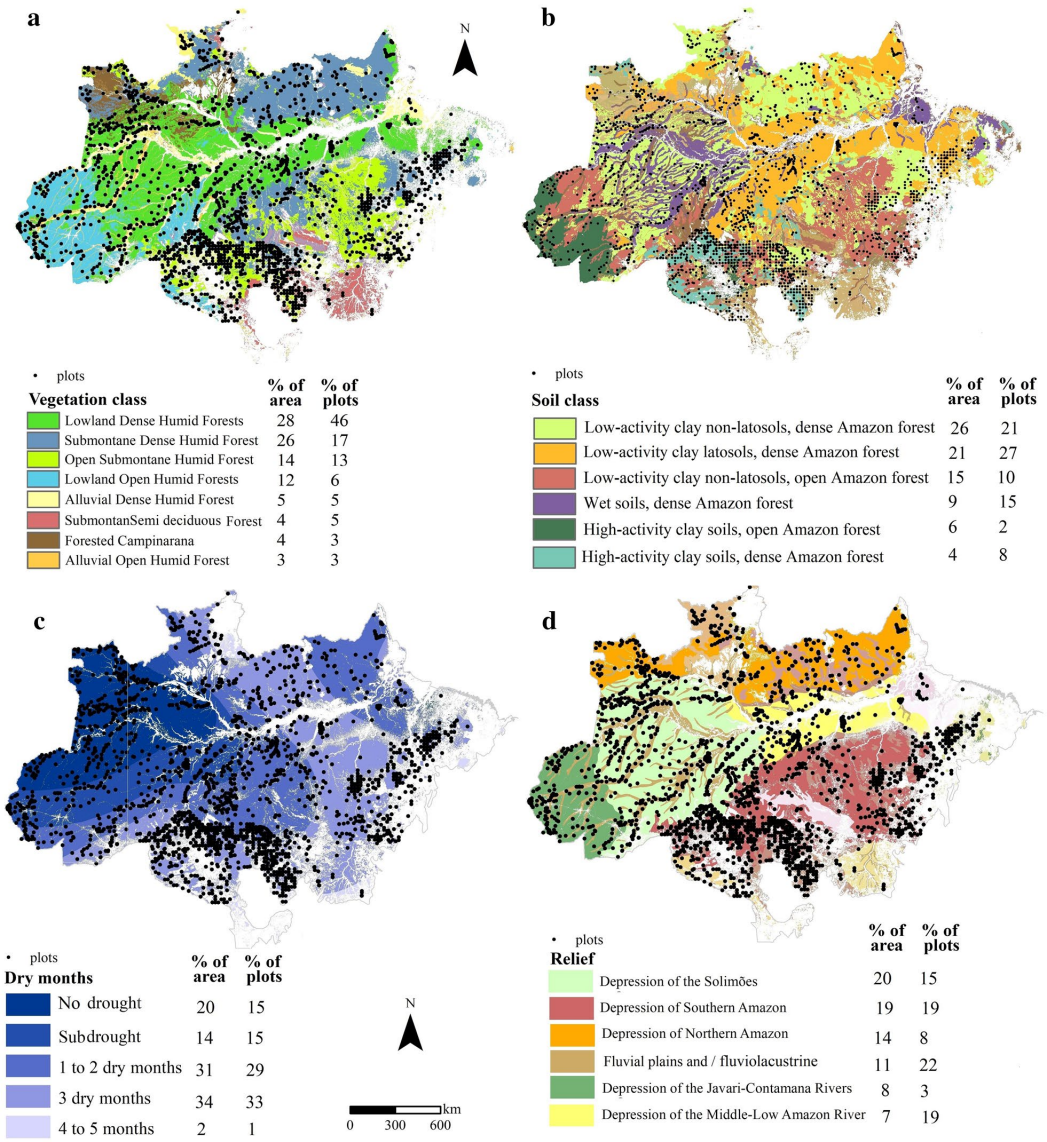
Environmental factor maps and forest inventory plots in the Brazilian Amazon. **a** Vegetation map with 28 classes [15]; **b** soil map with 42 classes [38]; **c** precipitation seasonality map, divided into 5 classes [40]; **d** topography map with 31 classes [39]. The complete legend is shown for the 6 largest classes, which comprise almost 80% of the total area and the total number of plots. The percentage of the area and number of plots for each class are shown. A detailed legend of names are provided in Additional file 1: Table S2, and the detailed areas and numbers of plots per class are provided in Additional file 1: Table S3

Crossing forest inventory plots and environmental factor maps, such as soil, vegetation, topography and precipitation (represented as dry months), few classes account for most of the plots of the Brazilian Amazon biome (Fig. 7). However, the represented classes usually account for the largest area.

Analyzing the vegetation types, lowland dense humid forests represent 28% of the area and comprise 44% of the plots; submontane dense humid forest represents 26%, and of the area and 17% of the plots; open submontane humid forest represents 14% of the area and 15% of the plots; and open ombrophilous lowland forest represents 12% of the area and 8% of the plots (Fig. 7a).

Only 4 of the 42 soil classes exhibit considerable numbers of plots. Low-activity clay non-latosols with dense Amazon forest (26% of the area) comprises 21% of the plots; low-activity clay latosols with dense Amazon forest (21% of the area) comprises 27% of the plots; low-activity clay non-latosols with open Amazon forest (15% of the area) has 10% of the plots; and wet soils with dense Amazon forest (9% of the area) has 15% of the plots. The remaining plots are spread out over the other 38 soil classes (Fig. 7b).

The classes for 1 to 2 dry months and 3 dry months represent the largest area, 31 and 34%, and comprise 29 and 34% of the plots, respectively. The other two classes with the largest amounts of rain represent 20 and 1% of the area, with each representing 15% of the plots (Fig. 7c). From a total of 31 topography classes, only 4 represent 65% of the plots. However, these 3 classes cover more than 70% of the Amazon (Fig. 7d).

## Discussion

The process of synthesizing and organizing the data of forest inventories, airborne LiDAR transects and AGB maps, is not trivial. Scientific literature usually mentions the location of the AGB data, but not always the project, the institution or network to which data belong. The social network analysis (SNA) was fundamental to organize and understand the AGB data and the relations between the stakeholders working in Brazilian Amazon forests.

The AGB data coverage shows that there is a lack of in situ information for large regions of the Amazon (Fig. 6). Excluding the RadamBrasil plots, by assuming that they are outdated (1973–1983), the area with no field data increases substantially. In this context, the NFI is a particularly important initiative. Depending on whether these data become available, the number of AGB plots will increase significantly, reaching 7000 systematically distributed plots in the Amazon biome, which will be remeasured regularly for long-term biomass monitoring (although the plot size is only 0.2 ha).

Gaining access to the data remains the largest challenge because most of these data are not currently publicly available (Fig. 6). INPA and NFI plots represent more than 50% of the total plots (Table 4). The problem regarding AGB data coverage will not be completely solved by implementing more plots if the information remains unavailable.

A lack of transparency or open data policies makes the analysis of uncertainty very difficult [68]. This analysis is required for monitoring and measurement, reporting and verification (MRV) in the context of the REDD+ national programs [7]. A consolidated and open-access AGB database is urgently needed to improve future National Communications and biomass mapping. In this context, funders play an important role in the AGB data distribution policy, requiring that the products of supported projects are free and openly available [7]. This could improve the uncertainty related to AGB data because the AGB data users’ feedback will help improve the datasets (e.g., the MapBiomas initiative [69]).

Local and regional efforts, such as PPBio, RAINFOR and SL, which gather data from numerous projects and networks (i.e., INPA, RAINFOR and TEAM) and make it public, are essential for monitoring AGB changes over time and the impacts of anthropogenic and climate change on carbon storage in the Amazon forest. These three stakeholders, which provide AGB data to the public, are the most connected in the SNA (Fig. 5 and Additional file 1: Table S1), showing the importance of improving collaborations and developing a consistent data sharing policy.

The SNA can be considered an initial attempt to map the AGB stakeholders connections (Fig. 5). The detected links between stakeholders do not necessarily imply synergy between them and, even more, do not imply resource optimization. Improving the synergies detection and analysis are fundamental for improving collaboration and enhancing financial aid. Federal public universities and national research institutes are fundamental players in the current network framework for generating AGB data. The communication between those groups should be improved, the data collection should be standardized, and, most importantly, the data distribution policy should be nationally established and linked to funding access.

The small coverage of the field plots (0.0013%) reveals the necessity of including and promoting national remote sensing approaches. Considering the ALS surveys, the sampled area covered increases to 0.197% of the Brazilian Amazon (0.014% for SL and 0.183% for EBA) (Table 4).

At the scale of the Brazilian Amazon, ASL data are improving forest mapping (Fig. 3), mainly through datasets collected by the SL and EBA projects [37, 54]. The goal of the EBA project with all the ALS data is to improve the AGB estimation of the Brazilian Amazon [37]. At a global scale, the Earth Explorer Biomass initiative [70] and the Global Ecosystem Dynamics Initiative (GEDI) mission promise to bring great contributions in the next 5 years [17]. Moreover, mapping environmental factors that influence the AGB estimation and distribution is highly recommended.

Despite the challenges at the field level, many AGB maps are available, although significant differences exist between the approaches used to generate those maps (Table 5). The reason for the observed differences in the quantity and distribution of AGB estimates (Fig. 4) is that each AGB map relies on different field data and different techniques for upscaling the AGB information to the map level [19, 23, 71].

**Table 5:** Approaches to mapping AGB of the Brazilian Amazon

AGB maps	Approaches to mapping carbon stocks	General description
Nogueira et al. [34]	Stratify and multiply	Assign an average AGB value to land cover/vegetation type map
MCT 2010 [15]		
Mitchard et al. [22]		
MCT [6]		
Saatchi et al. [18]	Direct remote sensing	Empirical models where remote sensing data are calibrated to field estimates
Baccini et al. [33]		
Avitabile et al. [35]		

The general approaches to mapping AGB are described according to Goetz et al. [72]

It is difficult to define AGB strata derived from environmental factors, such as vegetation, soil, precipitation and topography data. The interrelationships between these factors are not completely understood at the regional scale [18, 31, 73]. A better comprehension is urgently required to stratify and improve AGB estimations [74]. The implications of not considering stratification, based on either vegetation types, slope aspects, or the combination of both, for AGB estimations are the cost, time and work of establishing forest inventory plots and the high cost of acquiring airborne LiDAR transects due to the large area of the Brazilian Amazon biome. Thus far, there has been no consensus on AGB stratification in the Brazilian Amazon biome, which is why the NFI and the EBA project have opted for a systematic sampling instead of a stratified one. Our estimation of the number of AGB plots for each environmental factor map shows that the maps have many strata with a few large classes where most plots are located. The NFI, EBA and SL AGB data could be used to analyze which environmental factor map (or which strata) better represents AGB. Moreover, variance analyses of the AGB data (of maps and available plots) within each environmental factor map class should be considered in future studies.

## Conclusions

Several AGB stakeholders involved in forest inventories have different goals, protocols, and time frames for forest surveys; forest inventory data of the Brazilian Amazon remain unstandardized. Although some long-term relationships between the stakeholders exist, there is no standard protocol for distributing AGB data to ensure clarity, understandability and comparability. Research funding agencies have a very important role in establishing a clear sharing policy to make data free and open as well as in harmonizing the collection procedure. Such measures could have positive implications for National Communications, carbon mapping and REED+ activities.

The forest inventory plots coverage sampled a small fraction of the Brazilian Amazon forest carbon stocks. The NFI and airborne LiDAR data play an important role in filling gaps in the existing AGB data and updating the national scale information currently filled by the RadamBrasil dataset. Additionally, remote sensing data are crucial for covering continental areas, such as the Brazilian Amazon. It is essential to generate quality AGB data to monitor forest carbon and to understand the resilience of tropical forests facing deforestation, degradation, and climate change.

## Supplementary information

**Additional file 1: Table S1.** Table of social network analysis connections and acronyms from Fig. 5. **Table S2:** Detailed legend of Fig. 7. **Table S3:** Table of AGB plots per environmental factor maps in the Brazilian Amazon forest biome (using the 2014 forest mask); detailed legend is above in S2.

## Data Availability

The datasets supporting the conclusions of this article are included within the article and its Additional file 1.

## Abbreviations

AGB: aboveground biomass
ALS: airborne laser scanning
CCST: Earth System Science Center
EBA: improving biomass estimation methods for the Amazon, subproject 7 of Remote Sensing Environmental Monitoring of the Amazon Project
Embrapa: Brazilian Agricultural Research Corporation
INPA: National Institute of Amazon Research
INPE: National Institute for Space Research of Brazil
LBA: large-scale biosphere–atmosphere experiment in Amazonia
LiDAR: light detection and ranging
MRV: monitoring and measurement, reporting and verification
NFI: National Forest Inventory
PPBio: The Research Program for Biodiversity
PRODES: Deforestation Monitoring Program
SNA: social network analysis
SL: sustainable landscapes project
RAINFOR: Amazon Forest Inventory Network
REDD+: reducing emissions from deforestation and forest degradation and the role of conservation of forest carbon stocks, sustainable management of forests and enhancement of carbon stocks
TEAM: Tropical Ecology, Assessment, and Monitoring Network
TREES: Tropical Ecosystems and Environmental Sciences Laboratory
UNFCCC: United Nations Framework Convention on Climate Change
