# Supplementary material for: Evaluating spatial coverage of data on the aboveground biomass in undisturbed forests in the Brazilian Amazon
Source: Carbon Balance Manag. 2019 Sep 3;14:11. doi: 10.1186/s13021-019-0126-8 (PMC7226941; doi:10.1186/s13021-019-0126-8)
Supplement: Supplementary file 1 — Additional file 1: Table S1. Table of social network analysis connections and acronyms from Fig. 5. Table S2: Detailed legend of Fig. 7. Table S3: Table of AGB plots per environmental factor maps in the Brazilian Amazon forest biome (using the 2014 forest mask); detailed legend is above in S2. [file 13021_2019_126_MOESM1_ESM.docx]

# Additional file 1

##

## S1. Table of social network analysis connections and acronyms from Figure 5

The “Node” column is the name of the stakeholder; “Attribute” is the type of stakeholder; “Number of total connections” is the sum of the connections; “Connections” shows each connection between two stakeholders using their respective ID numbers. Acronyms are defined after the table.

| **ID** | **Nodes** | **Attribute** | **Number of total connections** | **% of connections** | **Connections** |
| --- | --- | --- | --- | --- | --- |
| 1 | UFRA | National universities | 1 | 0.4 | 14 15  15 58  58 40  58 36  40 16  40 17  40 19  40 20  40 18  40 21  40 22  40 23  40 24  40 25  58 25  36 24  36 23  41 38  41 36  42 41  42 28  42 36  42 38  56 54  14 56  55 39  15 37  15 36  15 48  15 51  15 26  15 38  15 39  15 33  39 28  14 46  14 54  57 38  35 36  53 38  53 42  54 39  30 39  27 39  43 1  43 2  43 3  43 4  43 5  43 44  43 55  43 37  53 30  51 53  51 31  53 12  53 13  31 35  31 11  31 53  31 58  31 54  31 46  31 36  31 44  35 48  35 34  35 10  35 31  35 53  35 50  35 54  35 49  35 51  35 47  35 60  35 46  35 9  35 8  35 7  35 6  41 66  44 56  44 60  44 51  44 58  44 54  44 61  44 62  44 63  44 64  44 65  44 67  44 8  44 68  45 44  45 46  51 30  69 54  28 5  28 58  28 63  28 4  28 68  28 70  28 54  28 37  28 57  28 52  28 7  28 15  28 41  28 3  28 55  28 38  28 64  28 64  28 72  28 71  28 73  28 74  28 59  58 75  28 75  28 19 |
| 2 | ESALQ | National universities | 1 | 0.4 |  |
| 3 | UFOPA | National universities | 3 | 1.1 |  |
| 4 | UFMT | National universities | 1 | 0.4 |  |
| 5 | UFAM | National universities | 1 | 0.4 |  |
| 6 | UFCG | National universities | 1 | 0.4 |  |
| 7 | USP | National universities | 2 | 0.7 |  |
| 8 | Leeds | International universities | 1 | 0.4 |  |
| 9 | UNICAMP | National universities | 2 | 0.7 |  |
| 10 | New Hampshire | International universities | 1 | 0.4 |  |
| 11 | Lancaster | International universities | 2 | 0.7 |  |
| 12 | Oxford | International universities | 1 | 0.4 |  |
| 13 | Exeter | International universities | 1 | 0.4 |  |
| 14 | RadamBrasil | Projects | 4 | 1.5 |  |
| 15 | LBA | Projects | 11 | 4.0 |  |
| 16 | CADAF | Projects | 1 | 0.4 |  |
| 17 | Tacape | Projects | 1 | 0.4 |  |
| 18 | Chichuá | Projects | 1 | 0.4 |  |
| 19 | Pronex | Projects | 1 | 0.4 |  |
| 20 | INCT-Madeiras | Projects | 1 | 0.4 |  |
| 21 | PPOPE | Projects | 1 | 0.4 |  |
| 22 | Piculus | Projects | 1 | 0.4 |  |
| 23 | Bionte | Projects | 2 | 0.7 |  |
| 24 | Jacaranda | Projects | 2 | 0.7 |  |
| 25 | Geoma | Projects | 2 | 0.7 |  |
| 26 | AMAZONICA | Projects | 1 | 0.4 |  |
| 27 | ESECAFLOR | Projects | 1 | 0.4 |  |
| 28 | PPBio | Main networks | 25 | 9.1 |  |
| 29 | FATE-Amazonia | Projects | 2 | 0.7 |  |
| 30 | Go Amazon | Projects | 2 | 0.7 |  |
| 31 | EBA | Projects | 9 | 3.3 |  |
| 32 | Amazon FACE | Projects | 2 | 0.7 |  |
| 33 | ATTO | Projects | 4 | 1.5 |  |
| 34 | Silva Carbon project | Projects | 1 | 0.4 |  |
| 35 | Sustainable Landscapes | Projects | 18 | 6.5 |  |
| 36 | EEST-INPA/ZF2 (Manaus-Amazonas) | Main sites | 9 | 3.3 |  |
| 37 | Humaita Forest Reserve (Acre) | Main sites | 2 | 0.7 |  |
| 38 | FLONA Tapajós (Santarem-Pará) | Main sites | 5 | 1.8 |  |
| 39 | Caxiuanã national forest (Belen-Pará) | Main sites | 6 | 2.2 |  |
| 40 | Amazon State Forest Inventory | Main networks | 11 | 4.0 |  |
| 41 | TEAM | Main networks | 7 | 2.5 |  |
| 42 | RAINFOR | Main networks | 22 | 8.0 |  |
| 43 | Redeflor | Main networks | 9 | 3.3 |  |
| 44 | National Forest Inventory | Main networks | 14 | 5.1 |  |
| 45 | FAO FRA | Institutions | 3 | 1.1 |  |
| 46 | National Forest Service | Institutions | 5 | 1.8 |  |
| 47 | TNC | Institutions | 1 | 0.4 |  |
| 48 | NASA | Institutions | 3 | 1.1 |  |
| 49 | IPAM | Institutions | 2 | 0.7 |  |
| 50 | IMAZON | Institutions | 1 | 0.4 |  |
| 51 | INPE | Institutions | 8 | 2.9 |  |
| 52 | IBAMA | Institutions | 1 | 0.4 |  |
| 53 | TREES | Institutions | 10 | 3.6 |  |
| 54 | Embrapa | Institutions | 7 | 2.5 |  |
| 55 | Emilio Goeldi Museum | Institutions | 4 | 1.5 |  |
| 56 | IBGE | Institutions | 3 | 1.1 |  |
| 57 | ICMBio | Institutions | 1 | 0.4 |  |
| 58 | INPA | Institutions | 10 | 3.6 |  |
| 59 | SDS | Institutions | 1 | 0.4 |  |
| 60 | US Forest Service | Institutions | 2 | 0.7 |  |
| 61 | UFRN | National universities | 1 | 0.4 |  |
| 62 | UFPR | National universities | 1 | 0.4 |  |
| 63 | UNIR | National universities | 1 | 0.4 |  |
| 64 | UNEMAT | National universities | 2 | 0.7 |  |
| 65 | IDEFLOR | Institutions | 1 | 0.4 |  |
| 66 | Conservation International | Institutions | 1 | 0.4 |  |
| 67 | UFAL | National universities | 1 | 0.4 |  |
| 68 | UFAC | National universities | 1 | 0.4 |  |
| 69 | Tropical Managed Forests Observatory | Main networks | 1 | 0.4 |  |
| 70 | UFRR | National universities | 1 | 0.4 |  |
| 71 | FIOCRUZ | Institutions | 1 | 0.4 |  |
| 72 | WWF | Institutions | 1 | 0.4 |  |
| 73 | FUNAI | Institutions | 1 | 0.4 |  |
| 74 | UEA | National universities | 1 | 0.4 |  |
| 75 | PDBFF | Project | 2 | 0.7 |  |


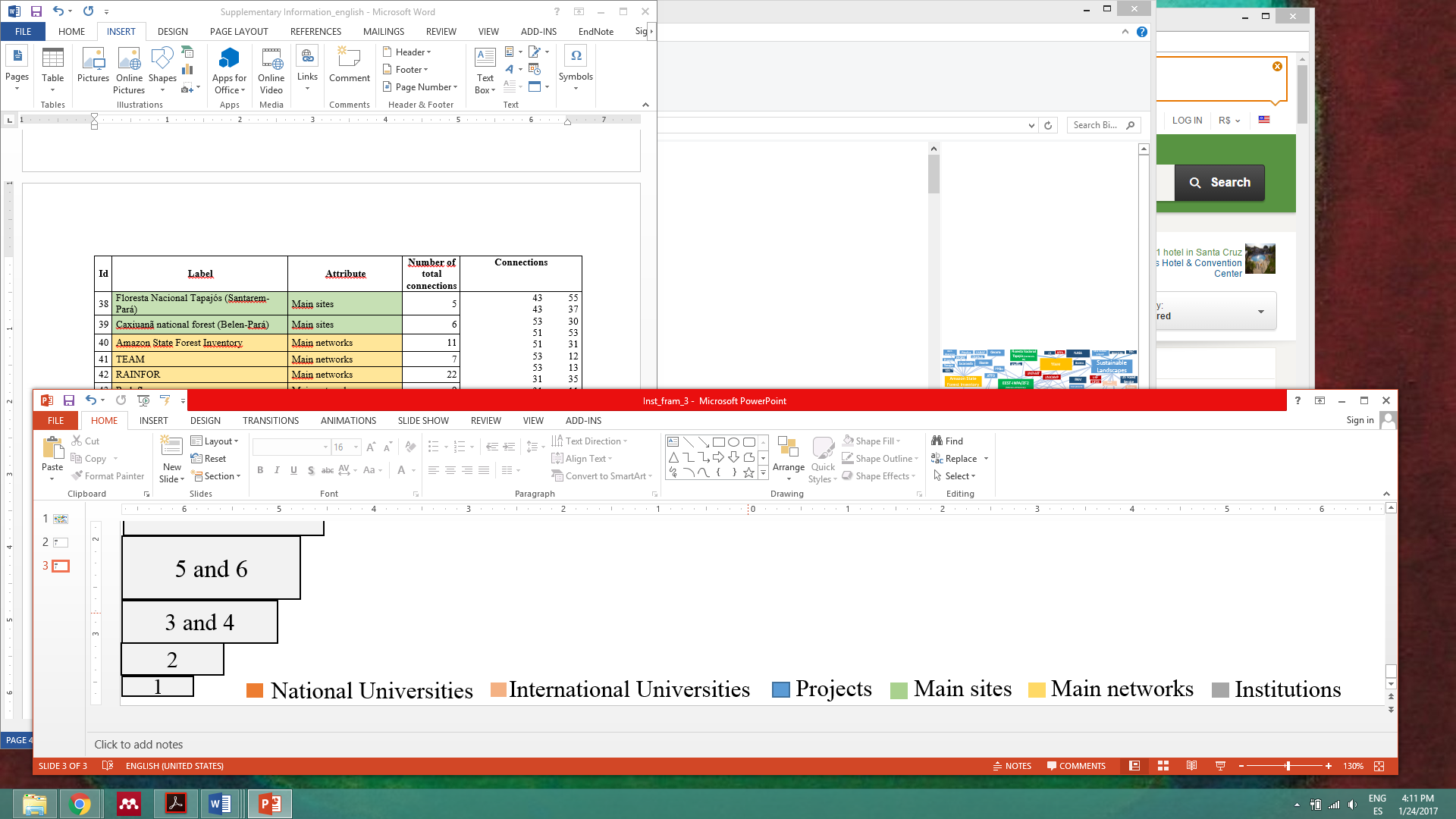


**Acronyms**

**Projects**

Amazon FACE Amazon Free-air CO_2_ Enrichment (FACE) Project

AMAZONICA Amazon Integrated Carbon Analysis Project

ATTO University of Leeds

Bionte Biomassa e Nutrientes

CADAF Carbon Dynamics of Amazonian Forests Project

Chichuá -

PDBFF

EBA Estimativa de Biomassa na Amazônia, subproject 7 of Remote Sensing Environmental Monitoring of the Amazon Project

ESECAFLOR Estudo Da Seca Na Floresta

FATE-Amazonia Fire-Associated Transient Emissions

Geoma -

Go Amazon Green Ocean Amazon

INCT-Madeiras Instituto Nacional de Ciência e Tecnologia de Madeiras da Amazônia

Jacaranda Pesquisas da Floresta Amazônica Brasileira

LBA Large-Scale Biosphere-Atmosphere Experiment in Amazonia

Piculus -

PPOPE -

Pronex Programa de Apoio a Núcleos de Excelência

RadamBrasil Projeto Radar da Amazônia

Silva Carbon Project -

Sustainable Landscapes -

Tacape -

**Networks**

Amazon State Forest Inventory Inventario Florestal Continuo

National Forest Inventory (NFI) Inventario Florestal Nacional

RAINFOR Amazon Forest Inventory Network

Redeflor Rede de Monitoramento da Dinâmica de Florestas na Amazônia Brasileira

PPBio Programa de Pesquisa em Biodiversidade (The Research Program for Biodiversity)

TEAM Tropical Ecology, Assessment, and Monitoring Network

Tropical Managed Forests Observatory -

**Institutions**

Conservation International -

Embrapa Empresa Brasileira de Pesquisa Agropecuária

Emilio Goeldi Museum Museu Paraense Emílio Goeldi

FAO FRA Global Forest Resources Assessments

FIOCRUZ Fundação Oswaldo Cruz

FUNAI Fundação Nacional do Índio

IBAMA Instituto Brasileiro de Meio Ambiente e Recursos Naturais Renováveis

IBGE Instituto Brasileiro de Geografia e Estatística

ICMBio Instituto Chico Mendes de Conservação da Biodiversidade

IDEFLOR Instituto de Desenvolvimento Florestal do Pará

IMAZON Instituto do Homem e Meio Ambiente da Amazônia

INPA National Institute of Amazon Research (Instituto Nacional de Pesquisas da Amazônia)

INPE National Institute for Space Research of Brazil (Instituto Nacional de Pesquisas Espaciais)

IPAM Instituto de Pesquisa Ambiental da Amazônia

NASA National Aeronautics and Space Administration

National Forest Service Serviço Florestal Brasileiro (SFB)

SDS Secretária de Estado do Meio Ambiente e Desenvolvimento Sustentável

TNC The Nature Conservancy

WWF World Wildlife Fund

**Sites**

Caxiuanã national Flona de Caxiuanã, Floresta Nacional de Caxiuanã

EEST-INPA/ZF2 Estação Experimental de Silvicultura Tropical (INPA)

Floresta Nacional Tapajós -

Humaita Forest Reserve -

**National Universities**

ESALQ Escola Superior de Agricultura Luiz de Queiroz

UFAC Universidade Federal do Acre

UFAL Universidade Federal do Alagoas

UFAM Universidade Federal do Amazonas

UFCG Universidade Federal de Campina Grande

UFMT Universidade Federal do Mato Grosso

UFOPA Universidade Federal do Oeste do Pará

UFPR Universidade Federal do Paraná

UFRA Universidade Federal Rural da Amazônia

UFRN Universidade Federal do Rio Grande do Norte

UFRR Universidade Federal de Roraima

UNEMAT Universidade do Estado de Mato Grosso

UNICAMP Universidade Estadual de Campinas

UNIR Universidade Federal de Rondônia

USP USP - Universidade de São Paulo

**International Universities**

Exeter University of Exeter,

Lancaster Lancaster University

Leeds University of Leeds

New Hampshire University of New Hampshire

Oxford University of Oxford

## S2. Detailed legend of Figure 7

### a) Vegetation Physiognomy map with 28 classes (MCT 2010)

**Phytophysiognomy Abbreviation**

Alluvial Open Humid Forest Aa

Lowland Open Humid Forest Ab

Open Submontane Humid Forest As

Lowland Deciduous Seasonal Forest Cb

Submontane Deciduous Seasonal Forest Cs

Alluvial Dense Humid Forest Da

Lowland Dense Humid Forests Db

Montane Dense Humid Forest Dm

Submontane Dense Humid Forest Ds

Alluvial Semideciduous Seasonal Forest Fa

Lowland Semideciduous Seasonal Forest Fb

Montane Semideciduous Seasonal Forest Fm

Submontane Semideciduous Seasonal Forest Fs

Wooded Campinarana La

Shrub Campinarana Lb

Forested Campinarana Ld

Wooded Grassy Campinarana Lg

Fluvial and/or Lacustrine-Influenced Vegetation Pa

Pioneering Formation of Fluviomarine Influence (Mangroves) Pf

Pioneering Formation of Marine Influence (Sand Banks) Pm

Mountain Refuge Rm

Wooded Savanna Sa

Forested Savanna Sd

Wooded Grassy Savanna Sg

Park Savanna Sp

Wooded Steppe Savanna Ta

Forested Steppe Savanna Td

Esthetic Park Savanna Tp

### b) Soil map with 42 classes (Bernoux et al. 1997), reflecting the intersection of soil class (V) and vegetation class (S)

**Vegetation category Soil Category**

V1 Open Amazon forest S1 HAC soils

V2 Dense Amazon forest S2 LAC latosols

V3 Atlantic forest S3 LAC non-latosols

V4 Seasonal deciduous forest S4 Sandy soils

V5 Seasonal semideciduous forest S5 Wet soils

V6 Mixed ombrophilous forest S6 Other soils

V7 Southern savanna

V8 Amazon savanna

V9 Savanna (Brazilian Cerrado)

V10 Southern Steppe

V11 Northeastern steppe

V12 Western steppe (Pantanal)

V13 Highland fields

V14 Areas of pioneer formations

V15 Woody oligotrophic vegetation

### c) Dry months with 5 classes (IBGE 2002)

1 to 2 dry months

3 dry months

4 to 5 dry months

No drought

Subdrought

### d) Topography with 31 classes (MMA 2002)

### Note: The Portuguese topographic names are first and the English translation. We translate from Portuguese to English the topographic terms, but the acronyms remain in Portuguese.

Pantanal of Guaporé River PRG

Depression of the River Araguaia/Pantanal DRAP

Depression of the River Xingu DRX

Depression of the upper Rivers Tocantins/Araguaia DARTA

Pantanal Mattogrossense PM

Residual plateaus of the Rivers Tocantins/Araguaia PRTA

Depression of Boa Vista DBV

Depression of the Rivers Negro/Branco DRNB

Escarps and Reverses of the Roraima Plateau ERPR

Mid-North Chapada CMN

Gurupi River ridges and hills CCRG

Lençóis Maranhenses LM

Marine plains, fluviomarine and/or fluviolacustrine environments PMFF

Seaboards TC

Chapada of Parecis CP

Depression of Southern Amazonia DAM

Depression of the Middle-Low Amazon River DMBRA

Depression of the Solimões DS

Depression of the upper Rivers Paraguay/Guaporé DARPG

Depression of the Javari-Contamana Rivers DRJC

Plateau of the Parecis PP

Plateaus and Sierras of the upper Rivers Paraguay/Guaporé PSARPG

Residual plateau of the Southern Amazon PRAM

Fluvial plains and/or fluviolacustrine environments PFF

Sierras of Cachimbo/Sucunduri SCS

Depression of the Lower Araguaia River DBRA

Depression of the Rivers Tocantins/Araguaia DMRTA

Mid-North depression DMN

Depression of the Northern Amazon DAS

Marginal plateaus to the Amazon River PMRA

Residual plateaus of the Northern Amazon PRAS

**Topographic names in Portuguese**

Pantanal do Rio Guaporé PRG

Depressão do Rio Araguaia/Pantanal DRAP

Depressão do Rio Xingu DRX

Depressão dos Altos Rios Tocantins/Araguaia DARTA

Pantanais Matogrossenses PM

Planaltos residuais dos Rios Tocantins/Araguaia PRTA

Depressão de Boa Vista DBV

Depressão dos Rios Negro/Branco DRNB

Escarpas e Reversos do Planalto de Roraima ERPR

Chapada do Meio-Norte CMN

Cristas e Colinas do Rio Gurupi CCRG

Lençóis Maranhenses LM

Planícies Marinhas, Fluviomarinhas e/ou Fluviolacustres PMFF

Tabuleiros Costeiros TC

Chapada dos Parecis CP

Depressão da Amazônia Meridional DAM

Depressão do Médio-Baixo Rio Amazonas DMBRA

Depressão do Solimões DS

Depressão dos Altos Rios Paraguai/Guaporé DARPG

Depressão dos Rios Javari-Contamana DRJC

Planalto dos Parecis PP

Planaltos e Serras dos Altos Rios Paraguai/Guaporé PSARPG

Planaltos Residuais da Amazônia Meridional PRAM

Planícies Fluviais e/ou Fluviolacustres PFF

Serras do Cachimbo/Sucunduri SCS

Depressão do Baixo Rio Araguaia DBRA

Depressão do Médios Rios Tocantins/Araguaia DMRTA

Depressão do Meio-Norte DMN

Depressão da Amazônia Setentrional DAS

Planaltos Marginais ao Rio Amazonas PMRA

Planaltos Residuais da Amazônia Setentrional PRAS

**S3. Table of AGB plots per environmental factor maps in the Brazilian Amazon forest biome (using the 2014 forest mask); detailed legend is above in S2**

| **Vegetation class (23)** | | | | **Dry months (52)** | | | | **Topography class (53)** | | | | **Soil class (50)** | | | |  |
| --- | --- | --- | --- | --- | --- | --- | --- | --- | --- | --- | --- | --- | --- | --- | --- | --- |
| **Classes** | **Plots** | **% of plots** | **% of area** | **Classes** | **Plots** | **% of plots** | **% of area** | **Classes** | **Plots** | **% of plots** | **% of area** | **Classes** | **Plots** | **% of plots** | **% of area** |  |
| AA | 103 | 2.68 | 2.64 | 1 to 2 dry months | 1133 | 29.43 | 30.72 | Chapada of Meio-Norte | 21 | 0.38 | 0.28 | V2S3 | 1169 | 20.93 | 25.99 |  |
| AB | 226 | 5.87 | 12.19 |  |  |  |  | Chapada of Parecis | 1 | 0.02 | 0.01 | V2S2 | 1647 | 29.48 | 21.27 |  |
| AS | 502 | 13.04 | 14.18 | 3 dry months | 1263 | 32.81 | 33.81 | Depression of Southern Amazon | 1155 | 20.68 | 18.78 | V1S3 | 515 | 9.22 | 15.06 |  |
| DA | 185 | 4.81 | 4.83 |  |  |  |  | Depression of Northern Amazon | 455 | 8.15 | 13.69 | V2S5 | 765 | 13.69 | 9.55 |  |
| DB | 1765 | 45.86 | 27.75 | 4 to 5 dry months | 52 | 1.35 | 1.94 | Depression of the Lower Araguaia River | 9 | 0.2 | 0.2 | V1S1 | 211 | 3.78 | 5.56 |  |
| DM | 24 | 0.62 | 1.18 |  |  |  |  | Depression of the Middle-Low Amazon River | 756 | 18.6 | 7.0 | V1S2 | 483 | 8.65 | 4.27 |  |
| DS | 635 | 16.50 | 25.62 | No drought | 815 | 21.17 | 19.83 | Depression of the Rivers Tocantins/Araguaia | 77 | 1.9 | 0.3 | V1S5 | 177 | 3.17 | 3.51 |  |
| FA | 7 | 0.18 | 0.09 |  |  |  |  | Mid-North Depression | 46 | 1.1 | 0.8 | V5S2 | 173 | 3.10 | 2.64 |  |
| FS | 200 | 5.20 | 3.94 |  |  |  |  | Depression of the Xingu River | 82 | 2.0 | 1.5 | V15S4 | 68 | 1.22 | 2.52 |  |
|  | 35 | 0.91 | 0.69 |  |  |  |  | Depression of the Solimões | 616 | 15.2 | 19.9 | V2S4 | 30 | 0.54 | 1.60 |  |
| LD | 115 | 2.99 | 3.74 |  |  |  |  | Depression of the upper Rivers Paraguay/Guaporé | 21 | 0.5 | 1.0 | Water | 67 | 1.20 | 0.97 |  |
| PA | 16 | 0.42 | 0.30 |  |  |  |  | Depression of the Javari-Contamana Rivers | 101 | 2.5 | 7.7 | V15S2 | 78 | 1.40 | 0.83 |  |
| LG | 1 | 0.03 | 0.08 |  |  |  |  | Depression of the Negro/Branco Rivers | 10 | 0.2 | 1.2 | V8S3 | 10 | 0.18 | 0.76 |  |
| Water | 12 | 0.31 | 0.29 |  |  |  |  | Escarpments and Reversos do Planalto of Roraima | 51 | 1.3 | 2.4 | V15S5 | 11 | 0.20 | 0.64 |  |
| RM | 4 | 0.10 | 0.06 |  |  |  |  | Pantanal of Guaporé River | 6 | 0.1 | 0.1 | V2S6 | 38 | 0.68 | 0.62 |  |
| SA | 2 | 0.05 | 0.36 |  |  |  |  | Plateau of the Parecis | 119 | 2.9 | 1.7 | V5S3 | 45 | 0.81 | 0.58 |  |
|  |  |  |  |  |  |  |  | Marginal plateaus of the Amazon River | 11 | 0.3 | 1.2 | V8S2 | 4 | 0.07 | 0.53 |  |
| SD | 15 | 0.39 | 1.48 |  |  |  |  | Residual Plateau of the Southern Amazon | 101 | 2.5 | 3.9 | V5S4 | 19 | 0.34 | 0.53 |  |
| SP | 1 | 0.03 | 0.13 |  |  |  |  | Residual Plateaus of the Northern Amazon | 48 | 1.2 | 3.3 | V8S4 | 2 | 0.04 | 0.49 |  |
| CS, FM, LB, PF, SG, TD | | 0 | < 0.5 |  |  |  |  | Residual plateaus of the Rivers Tocantins/Araguaia |  | 1 | 0.0 | V2S1 | 25 | 0.45 | 0.48 |  |
|  |  |  |  |  |  |  |  | Fluvial plains and/or fluviolacustres | 874 | 21.5 | 10.8 | V1S4 | 15 | 0.27 | 0.45 |  |
|  |  |  |  |  |  |  |  | Marine Plains, Fluvial | 13 | 0.3 | 1.3 | V5S5 | 4 | 0.07 | 0.24 |  |
|  |  |  |  |  |  |  |  | Saws of Cachimbo | 25 | 0.6 | 2.1 | V14S5 | 10 | 0.18 | 0.21 |  |
|  |  |  |  |  |  |  |  | Seal boards | 3 | 0.1 | 0.9 | V8S5 | 5 | 0.09 | 0.18 |  |
|  |  |  |  |  |  |  |  |  |  |  |  | V5S1 | 8 | 0.14 | 0.18 |  |
|  |  |  |  |  |  |  |  |  |  |  |  | V9S2 | 2 | 0.04 | 0.15 |  |
|  |  |  |  |  |  |  |  |  |  |  |  | V4S3 | 0 | 0.00 | 0.05 |  |
|  |  |  |  |  |  |  |  |  |  |  |  | V6S5 | 0 | 0.00 | 0.03 |  |
|  |  |  |  |  |  |  |  |  |  |  |  | V13S3 | 2 | 0.04 | 0.02 |  |

**Topography classes in Portuguese**

Chapada do Meio-Norte

Chapada dos Parecis

Depressão da Amazônia Meridional

Depressão da Amazônia Setentrional

Depressão do Baixo Rio Araguaia

Depressão do Médios Rios Tocantins/ Araguaia

Depressão do Médio-Baixo Rio Amazonas

Depressão do Meio-Norte

Depressão do Rio Xingu

Depressão do Solimões

Depressão dos Altos Rios Paraguai/ Guaporé

Depressão dos Rios Javari-Contamana

Depressão dos Rios Negro/ Branco

Escarpas e Reversos do Planalto de Roraima

Pantanal do Rio Guaporé

Planalto dos Parecis

Planaltos Marginais ao Rio Amazonas

Planaltos Residuais da Amazônia Meridional

Planaltos Residuais da Amazônia Setentrional

Planaltos residuais dos Rios Tocantins/ Araguaia

Tabuleiros Costeiros

Planícies Fluviais e/ou Fluviolacustres

Planícies Marinhas, Fluviomarinhas

Serras do Cachimbo
